# A new *Mycobacterium leprae* dihydropteroate synthase variant (V39I) from Papua, Indonesia

**DOI:** 10.1016/j.heliyon.2019.e01279

**Published:** 2019-03-07

**Authors:** Yustinus Maladan, Hana Krismawati, Hotma Martogi Lorensi Hutapea, Antonius Oktavian, Ratu Fatimah, Widodo

**Affiliations:** a Institute of Health Research and Development Papua, Ministry of Health, Jl. Ahmad Yani no 48, Jayapura, Indonesia; b Graduate School of Science and Technology, Kumamoto University, 2-39-1 Kurokami, Chuo-ku, Kumamoto, 860-8555, Japan; c Biology Department, Faculty of Mathematics and Natural Sciences, Brawijaya University, Jl. Veteran, Malang, Indonesia

**Keywords:** Molecular biology, Bioinformatics

## Abstract

Indonesia had the third highest number of new leprosy cases worldwide in 2017. This disease is still prevalent in Papua province, where the number of new cases in 2014 (3.0 cases per 10,000 people) is considered highly endemic and is well above the World Health Organization's (WHO) cutoff of <1 new case per 10,000 people. Since 1995, the WHO has supplied Papua province with a multi-drug therapy (MDT) in which multibacillary (MB) patients are treated with rifampicin, clofazimine, and dapsone and paucibacillary (PB) patients are treated with rifampicin and dapsone. Recent published data on global drug resistance reported cases of dapsone resistance in relapsed and newly diagnosed cases in Indonesia during this period. The detection of specific point mutations in *folP1* that encode dihydropteroate synthases (DHPS) is used exclusively to identify dapsone resistant strains of *Mycobacterium leprae*. The purpose of this study was to test for the presence of *folP1* mutations in *M. leprae* strains isolated from patients residing in Papua Island, Indonesia who responded less effectively to dapsone. This study identified a *folP1* point mutation that changed a valine (V) residue at amino acid position 39 (from the N-terminus) to isoleucine (I) (V39I) of DHPS. The V39I variant is located within an α-helix motif that may not much affect its structure. Molecular docking analysis indicated that the binding affinity of the V39I variant was slightly reduced as compared to the wildtype of DHPS. The decreasing of affinity may have a consequence of increasing inhibition constants (Ki) of dapsone on the variant V39I of DHPS. The data suggest that the DHPS V39I variant might cause less sensitive to dapsone. However, *in vivo* studies (e.g., mouse footpad model) are needed to confirm the effect of this DHPS variant on dapsone therapy.

## 1 Introduction

Indonesia has the third highest rate of new cases of leprosy, with 15,910 cases diagnosed in 2017, as compared with India (126,164) and Brazil (26,875) [1]. These three countries collectively account for just over 80% of the global leprosy disease burden. Based on the number of disability people affected by leprosy in 2013, Papua province had the highest number of leprosy cases (26.88), following by Aceh province (18.62), and Papua Barat province (17.72) [2, 3]. This disease is still prevalent in Jayapura City, Papua province, where the disease burden is considered to be high [4]. The number of new cases in Papua province in 2014 (3.0 per 10,000 people) is considered to be highly endemic and exceeds the WHO's cutoff of <1 new case per 10,000.

Leprosy affects skin, mucus membranes, and nerves [5]. Dapsone is a bacteriostatic therapy component of MDT. Leprosy patients take dapsone nearly everyday during yearlong therapy for MB and 6-month therapy for PB. The target of dapsone is DHPS, a key enzyme in the folate biosynthesis pathway in bacteria, including *M. leprae*. This enzyme is encoded by the *folP1* gene [6]. The mechanism of dapsone resistance was described by William and Gillis, which is caused by mutations within the drug resistance determining region (DRDR) of the *folP1* gene [7]. Missense mutations located at codon 53 (T53I, T53R, and T53A) and codon 55 (P55R, P55L) of *folP1* were also described by Cambau and Carthagena [8]. You et al. reported a previously undescribed polymorphism in *folP1* consisting of a C to T substitution at nucleotide 153 (P55S) in a strain isolated in Korea [9]. Detection of point mutations in *folP1* is considered to be the sole basis of identifying dapsone resistant strains of *M. leprae* [10]. Dapsone-resistant *M. leprae* isolates commonly contain point mutations in *folP1* at codon positions 53 or 55 [6, 8, 11]. The most frequently detected variation associated with dapsone resistance is a CCC→CTC codon change at codon position 55, resulting in the substitution of leucine (L) for proline (P) (P55L) [7]. This mutation decreases the effectiveness of dapsone therapy.

Until now, it has not been possible to culture *M. leprae* in axenic media and this species grows very slowly *in vivo*. The mouse footpad model is an essential method for evaluating drug-resistance of *M. leprae* strains, but this method is very costly and requires several months to over one-year to complete. DNA sequencing and protein modelling are feasible approaches for assessing drug resistance mediated by mutations in the target gene [12, 13]. Likewise, bioinformatics simulation (*in silico*) can be used to study drug resistance resulting from amino acid changes in the target protein. This method is relatively accurate, rapid, and cost-effective as compared to *in vitro* and *in vivo* methods and can be used to perform computational studies of drug resistance [14, 15]. Previous molecular docking studies have successfully predicted the location of ligand binding sites in protein receptors [16].

Since 1995, the WHO has supplied MDT to all countries with a significant leprosy burden. The MB form of the disease is treated with three antibiotics: rifampicin, clofazimine, and dapsone whereas the PB form is treated with a combination of rifampicin and dapsone [17]. Unfortunately, the MDT program in Papua and West Papua has been hindered by challenges including poor awareness of the program, geographical barriers that prevent access to health facilities, and negative paradigms of drugs held by residents.

The objective of this study was to evaluate *folP1* mutations in *M. leprae* strains isolated from patients residing in Papua Island, Indonesia that were less responsive to dapsone treatment. The binding affinity of the DHPS variant protein for dapsone was examined by *in silico* analysis.

## 2 Methods

### 2.1 Identification of *folP1* mutations

This research was approved by the Ethics Committee of the National Institute of Health Research and Development, Ministry of Health, Republic of Indonesia. Study samples obtained from new patients, relapsed patients, and patients that were less sensitive to dapsone treatment. A total of 100 patients were included in the study. Patient samples were subjected to DNA extraction using a QIAamp DNA Mini Kit (Qiagen). Purified DNA samples were used in polymerase chain reactions (PCRs) designed to amplify *folP1* gene, which is used primers included WHOF1: 5′-GCAGGTTATTGGGGTTTTGA-3’ (forward primer) and WHOF2: 5′-CCACCAGACACATCGTTGAC-3’ (reverse primer). A touchdown PCR method was performed, preheating was done at 98 °C for 2 minutes, followed by 5 cycles of 98 ^o^C for 20 seconds, 60 ^o^C to 56 ^o^C with decrement 1 ^o^C per cycle for 30 seconds, and 72 ^o^C for 20 seconds. Further cycle was done at 98 °C for 20 seconds, 55 °C for 30 seconds, and 72 °C for 30 seconds for p 40 times, with a final extension at 72 °C for 5 minutes. PCR products were purified by ExoSAP-IT™ PCR Product Cleanup (Thermo Fisher Scientific) and were then subjected to DNA sequencing using 2.5X BigDye Terminator v3.1 Ready Reaction Mix (4 μL), 5X BigDye Terminator buffer v1.1/v3.1 (4 μL), template DNA (1 μL), and nuclease-free water (7 μL). pGEM-3Zf and sequencing primer -21 M13 were used as a positive control. Sequencing was conducted on a 3500 Series Genetic Analyzer (Applied Bosystems). *folP1* mutations were identified by processing the sequencing results in GenBank.

### 2.2 Analysis of *folP1* and dihydropteroate synthase variants

*The folP1* sequences isolated from the various *M. leprae* strains were aligned with the sequence of the *folP1 gene* (GenBank accession number NC_002677.1) using BioEdit (http://www.mbio.ncsu.edu/bioedit/bioedit.html). Additionally, translated amino acid sequences of the DHPS variants were aligned with non-redundant DHPS sequences (WP_010907608.1) of *M. leprae* Br4923 [18], and other variants that reported by Kai M. et al., BAA84081.1, BAA84079.1, and BAA84076.1 [19] using BioEdit [20].

### 2.3 Homology modeling of dihydropteroate synthase

The DHPS amino acid sequence (accession number NP_301284.1) was downloaded from GenBank (https://www.ncbi.nlm.nih.gov/) in FASTA format. The 3D structure of DHPS was derived using I-TASSER, starting from the structure templates identified by LOMETS from the PDB library. LOMETS is a meta-server threading approach that contains multiple threading programs, where each threading program can generate tens of thousands of template alignments [21, 22]. The position of the ligand binding site was predicted by COFACTOR and COACH. COFACTOR derives protein function (ligand-binding sites, EC, and GO) based on structural comparisons and protein-protein networks. COACH is a meta-server approach that combines multiple annotation results (on ligand-binding sites) from the COFACTOR, TM-SITE, and S-SITE programs [23]. The T53I, V39I, P55L, and T53A variants of DHPS were constructed using FoldX [24], based on the wild-type DHPS structure. The structures were repaired and protein stability was evaluated using foldX under the Yasara viewer.

### 2.4 Preparation of drug molecules

The structure of dapsone was downloaded from the Pubchem database (ID 2955) in format sdf file, available at https://pubchem.ncbi.nlm.nih.gov/compound/2955. The energy of the dapsone molecule was minimized based on uff force field with total number of steps were 200 and energy difference less than 0.1. The sdf file of dapsone was converted to the pdbqt format using Open Babel software [25] that ready for docking analysis.

### 2.5 Molecular docking

Docking analysis was performed by AutoDock using a Lamarckian genetic algorithm [26], which is integrated with a PyRx application (https://sourceforge.net/projects/pyrx/) [27]. Docking results were visualized using PyMol 1.8.6 and Discovery Studio 2016 Client. 3D structures of dapsone inhibitors were downloaded from the NCBI database (CID 2955). A grid of 181, 180, 195 points in x, y, and z direction which grid spacing of 0.375 Å was built. The grid center was 59.433, 63.232, and 58.536 points in x, y, and z, respectively. The ligands with the lowest of binding energy were selected as the resultant complex structures. The structure molecules were visualized by Yasara view [28] or Discovery studio [29].

## 3 Results

**Fig. 1:**
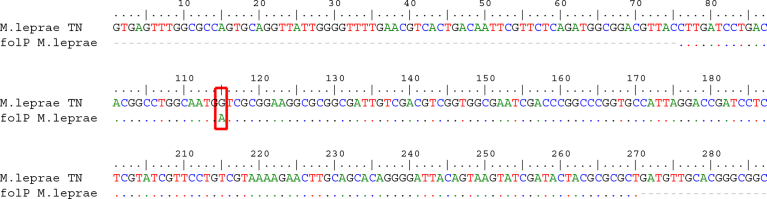
Alignment of the *folP1* sequence isolated from Papua province patients with the *M. leprae folP1* sequence (GenBank accession number NP_301284.1). The G to A transition is indicated.

**Fig. 2:**
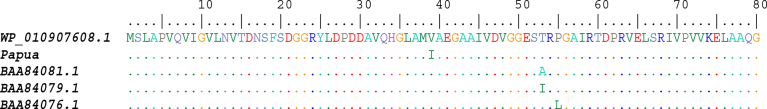
Comparison of the DHPS protein sequence of *M. leprae* from Papua province with other dapsone-resistant reference strains. The mutation in the Papua province *M. leprae* strain is V39I.

*The partial sequence of folP1* gene were generated by PCR from 50 of the 100 West Papua patient samples that were subsequently subjected to Sanger sequencing. Mutations were identified by aligning sequencing data with the sequence of the *folP1* gene sequence from GenBank. A missense mutation of *folP1* at nucleotide position 115 (guanine (G) to adenine (A)) that changed the corresponding amino acid from a valine to an isoleucine (Fig. 1) was identified in one patient sample. The position of this V39I mutation is different than the codon 53 and codon 55 mutations commonly found in dapsone-resistant strains (Fig. 2). The patient from which this *folP1* mutation was identified was a 15-year-old boy originally from Teluk Bintuni district, Papua province. He was diagnosed with the PB form of leprosy and started therapy in April 2015, but he did not accomplish and re-started therapy in 2017.

**Fig. 3:**
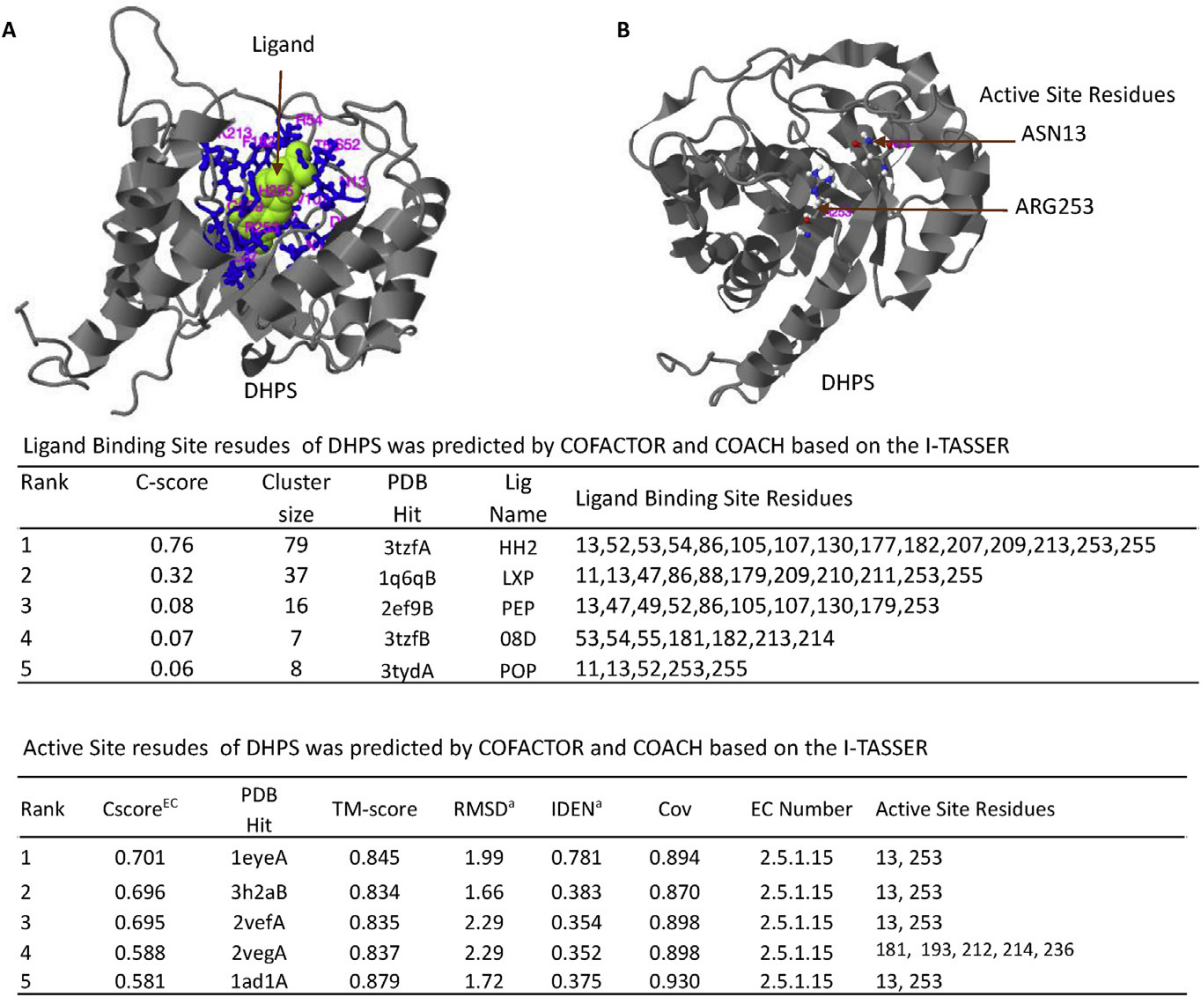
The ligand binding site of DHPS (A) and active site residues (B) of DHPS, predicted by I-TASSER. The rank and prediction scores are shown in the bottom panel of the table. **C-score** is the confidence score of the prediction in which a higher score indicates a more reliable prediction. **Cluster size** is the total number of templates in a cluster. **Lig Name** is the name of the possible binding ligand. **Cscore**^EC^ is the confidence score for the EC number prediction, where a higher score indicates a more reliable EC number prediction. **TM-score** is a measure of global structural similarity between the query and the template protein. **RMSD** is the root-mean-square deviation between residues that were structurally aligned by TM-align. **IDEN** is the percent sequence identity in the structurally aligned region. **Cov** represents the coverage of global structural alignment and is equal to the number of structurally aligned residues divided by the length of the query protein.

The 3D structure of the DHPS protein was modeled by the I-TASSER software using threading. The I-TASSER software used COFACTOR and COACH to predict ligand binding positions and active sites based on the modeling results. This analysis estimated the ligand binding site is comprised of 15 amino acids, one of which is amino acid number 53. The DHPS active site was estimated to be similar to the active site of DHPS from *Mycobacterium tuberculosis* (PDB ID:1eye) at amino acid residue position 13 and 253 (Fig. 3).

**Fig. 4:**
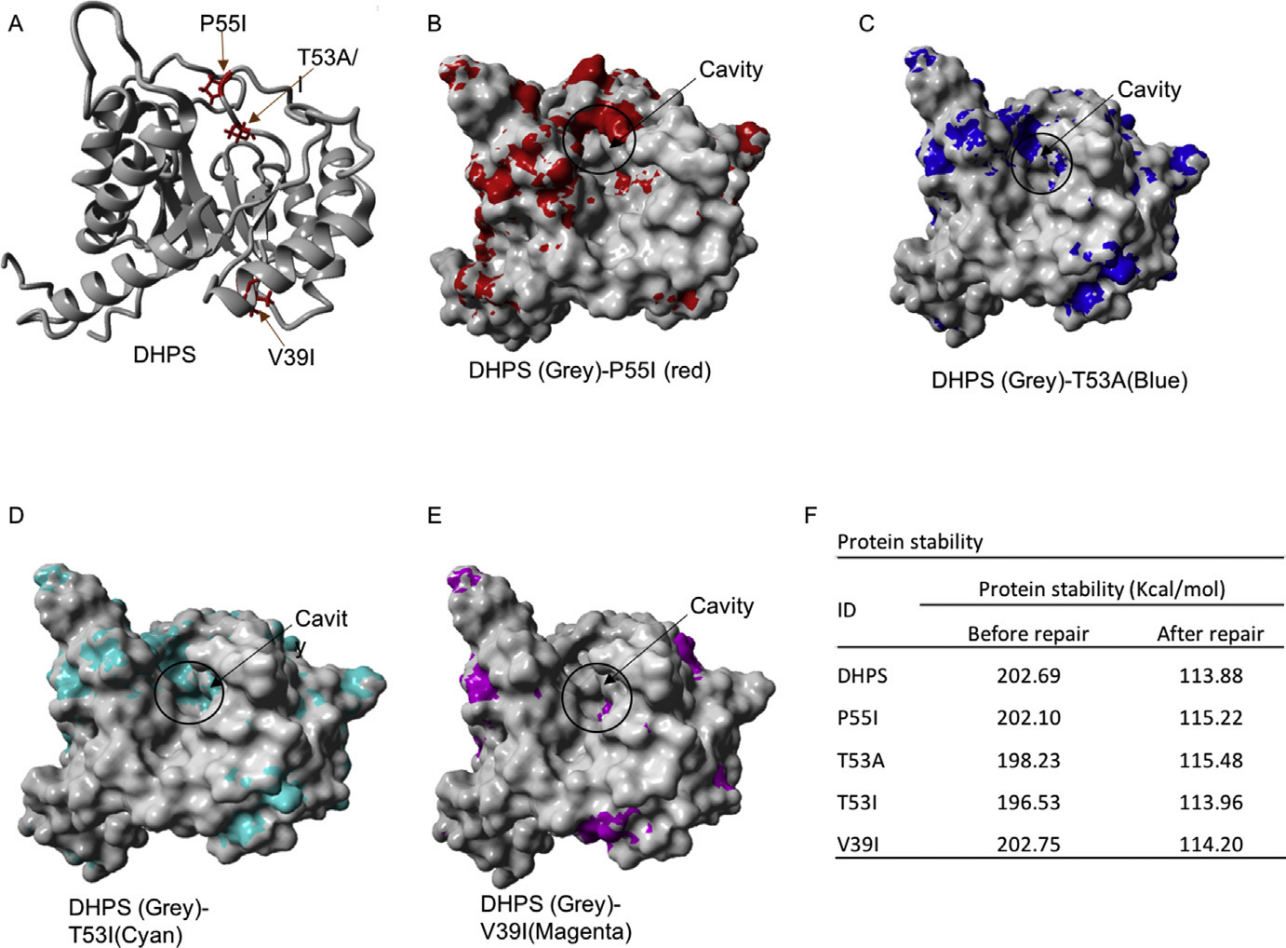
The position of amino acid changes in DHPS (A), which significantly changed the cavity surface of P55I (B) followed by T53A (C), T53I (D), and T53I variants has just least change compared to wildtype (D). Amino acid variations also changed the energy stability of DHPS (F).

FOLDX was used to construct DHPS structures containing P55I, T53A, T53I, and V39I mutations, using the 3D structure derived from modeling as a template. The 3D structure of all DHPS variants was repaired and the energy stability of each variant was calculated using FOLDX. Protein repair resulted in better protein stability (Fig. 4 F). The P55I mutation resulted in the most significant changes to the cavity surface of DHPS protein, followed by T53A and T53I. The V39I mutation imparted very little change to the cavity surface of DHPS (Fig. 4). These cavity surface changes would likely affect the binding interactions between DHPS and dapsone. To test dapsone binding by DHPS, molecular docking was performed using Autodock in the PyRx software.

The results of the docking analysis showed that the amino acid changes in DHPS affected binding to dapsone. The predicted binding energy between dapsone and wild-type DHPS was different than the binding energy of the variants. The P55I variant has the highest binding energy, followed by T53A, T53I, and V39I. The changes in binding energy affect the inhibitory constant (Ki) that may alter the activity of dapsone. Of the DHPS variants, the properties of V39I were most similar to wildtype, with a binding energy of -6.26 kcal/mol and a Ki of 25.78 uM (Table 1).

**Table 1:** Dapsone and DHPS binding energy derived by autodock prediction.

ID	Binding Energy (Kcal/mol)	Inhibition Constant (uM)	Intermol Energy (Kcal/mol)	Internal Energy (Kcal/mol)	Torsional Energy (Kcal/mol)	Unbound Energy (Kcal/mol)
DHPS	-6.28	24.93	-7.47	-0.29	1.19	-0.29
P55I	-5.53	88.40	-6.72	-0.31	1.19	-0.31
T53A	-5.90	47.34	-7.09	-0.26	1.19	-0.26
T53I	-6.20	28.53	-7.39	-0.29	1.19	-0.29
V39I	-6.26	25.78	-7.45	-0.28	1.19	-0.28

**Fig. 5:**
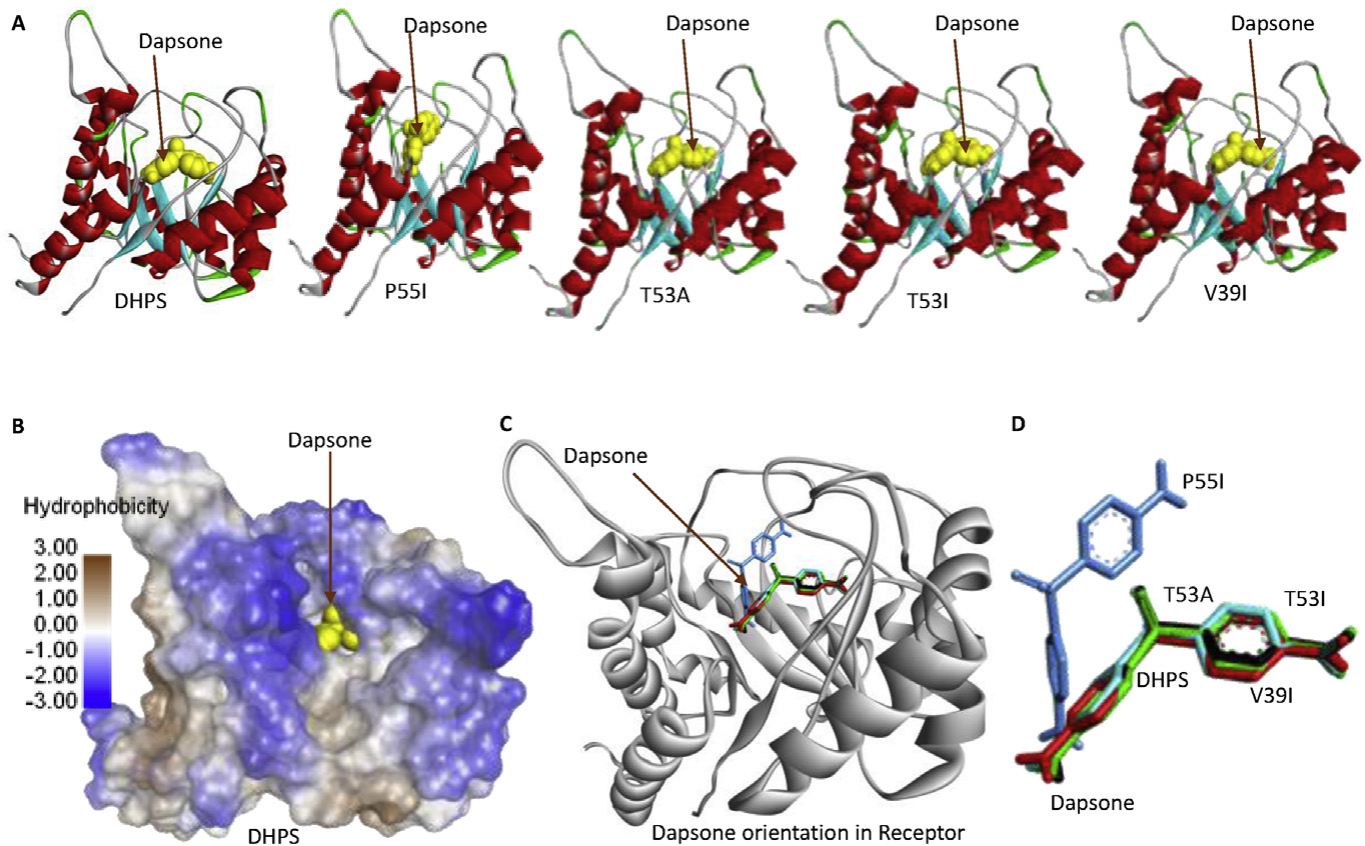
Dapsone binding orientation to wild-type DHPS and its variants (A). Dapsone binds to the cavity in the middle of the hydrophobic cavity (B and C). Dapsone's binding orientation to the P55I variant is different as compared to the other three variants (T53A, T53I, and V39I) and wild-type DHPS (D).

The change in binding energy of dapsone to DHPS is thought to change the binding orientation of dapsone to DHPS. Next, we analyzed the orientation of dapsone binding to each DHPS variant, which showed that the orientation of dapsone binding to T53A, T53I, and V39I was similar to wild-type, but was different with P55I (Fig. 5). However, dapsone is located in the same hydrophobic cavity for all of DHPS variants. Changing the orientation of dapsone binding to P55I is thought to disrupt dapsone's ability to inhibit DHPS activity that results in resistance. While the orientation of dapsone binding to the V39I variant is similar to wild-type, there is an increase in V39I's binding energy that it is thought to reduce the sensitivity of V39I to dapsone.

**Fig. 6:**
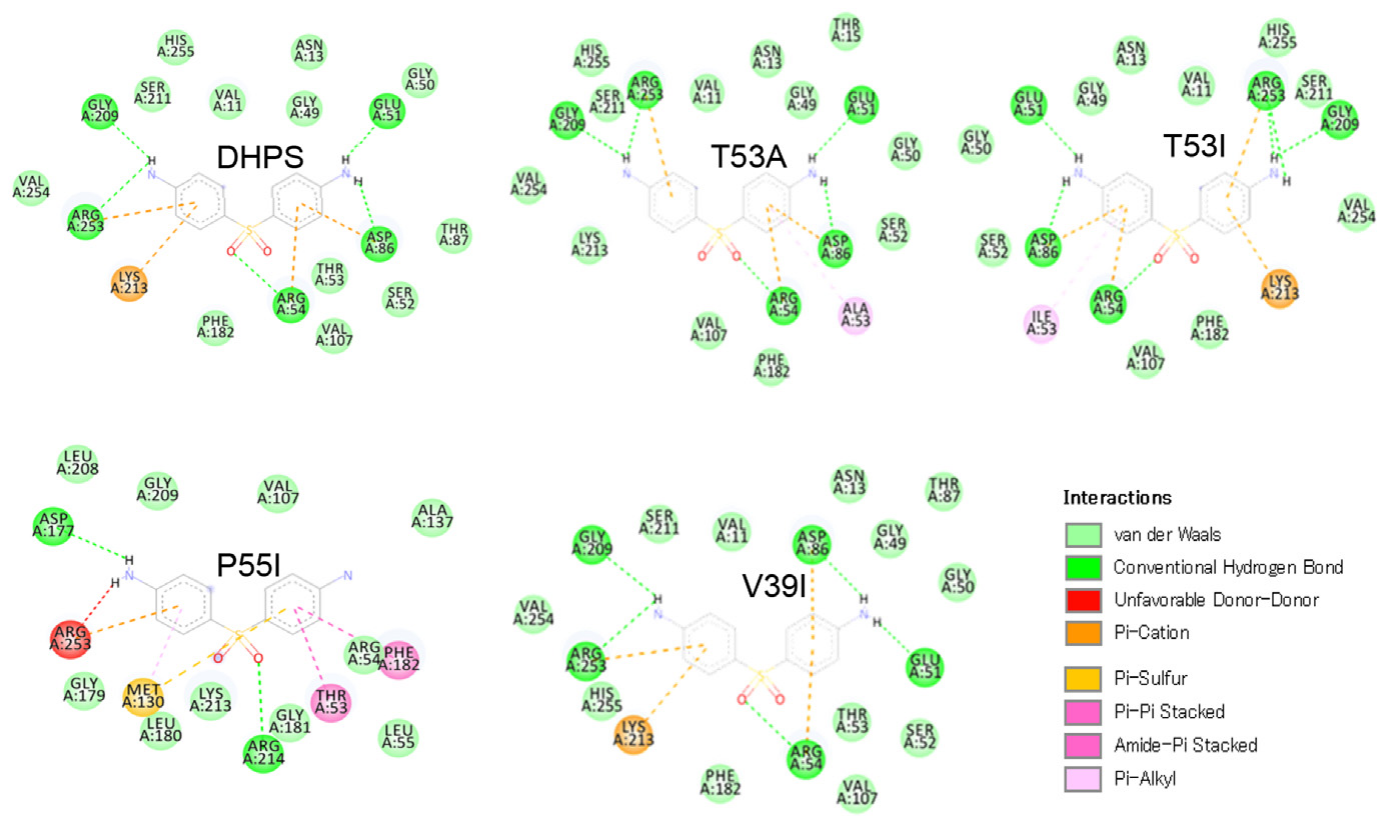
The chemical bonds formed between dapsone and DHPS, including the four variants: P55I, T53A, T53I, and V39I. Amino acid substitutions alter the chemical bonding pattern. P55I significantly alters the bonding pattern with dapsone as compared to wildtype, followed by T53A, T53I, and V39I.

Next, we analyze the chemical bonds formed between dapsone and wild-type DHPS and its variants. The results of this analysis indicate that dapsone forms hydrogen bonds with wild-type DHPS and the T53A, T53I, and V39I variants at amino acid residues Arg253, Arg54, Asp86, and Glu51 (Fig. 6 and Table 2). The hydrogen bond formed between dapsone and Arg253 is thought to inhibit DHPS enzymatic activity because Arg253 is part of the enzymatic active site. Dapsone does not form hydrogen bonds with Arg253 of the P55I variant, which is predicted to reduce the effectiveness of dapsone as an inhibitor of DHPS. The V39I mutation abolishes only one hydrophobic bond between dapsone and DHPS on Val254, which has only a minimal effect on binding energy. However, the binding affinity change is predicted to reduce the binding of dapsone to DHPS, thereby reducing the sensitivity of *M. leprae* to dapsone (Fig. 6 and Table 2).

**Table 2:** The type of chemical bonds between dapsone and wild-type and variant DHPS.

NO	Interaction	DHPS	P55I	T53A	T53I	V39I
1	Hydrogen Bond	Arg253	Arg214	Arg253	Arg253	Arg253
		Arg54	Asp177	Arg54	Arg54	Arg54
		Asp86		Asp86	Asp86	Asp86
		Glu51		Glu51	Glu51	Glu51
		Gly209		Gly209	Gly209	Gly209
2	Van der Waals	Asn13	Ala137	Asn13	Asn13	Asn13
		Gly49	Arg54	Gly49	Gly49	Gly49
		Gly50	Gly179	Gly50	Gly50	Gly50
		His255	Gly181	His255	His255	His255
		Phe182	Gly209	Lys213	Phe182	Phe182
		Ser211	Leu180	Phe182	Ser211	Ser211
		Ser52	Leu208	Ser211	Ser52	Ser52
		Thr53	Leu55	Ser52	Val107	Thr53
		Thr87	Lys213	Thr15	Val11	Thr87
		Val107	Val107	Val107	Val254	Val107
		Val11		Val11		Val11
		Val254		Val254		
3	Unfavourable donor-Donor	-	Arg253	-	-	-
4	Pi-Cation	Lys213	-	-	Lys213	Lys213
5	Pi-Sulfur	-	Met130	-	-	-
6	Pi-Akyl	-	-	Ala53	Ile53	-
7	Pi-Pi Stacked	-	Phe182	-	-	-
			Thr53			

## 4 Discussion

Recently published data on global drug resistance to rifampicin (*rpoB*), dapsone (*folP1*), and ofloxacin (*gyrA*) detected by the WHO surveillance network in 19 endemic countries for the period 2009–2015 using PCR to detect mutations in DRDRs included data from Indonesia [30]. This study found that 3/91 (3.3%) cases of rifampicin resistance and 3/70 (4.3%) cases of dapsone resistance were reported during this period in Indonesia among relapsed and newly diagnosed cases, compared with 87 cases of dapsone resistance detected among 1,932 individuals (4.5%) [30]. Moreover, we found a new variant G115A of *folP1* gene that changed amino acid number 39 from valine to isoleucine (V39I) of DHPS protein. This mutation is located in the DRDR of the *folP1* gene. However, the effect of this mutation has not been reported. Therefore, we performed a molecular docking analysis to investigate DHPS variants that would change the binding affinity to dapsone. The results of the analysis showed that the V39I variant has slightly reduced binding affinity as compared to wild-type. The V39I mutation increased the Ki of dapsone. The data suggested that the V39I variant may reduce sensitivity to dapsone.

In this study, we also analyzed the interaction between dapsone and the P55L, T53I, and T53A variants of DHSP using molecular docking, which are known to contribute to the resistance of *M. leprae* to dapsone. The molecular docking analysis among known variant to the dapsone for providing basic information to understanding the possible effects of the V39I mutation on dapsone by using same method analysis. The results of this analysis indicate that the orientation of dapsone binding was altered by the P55I mutation, which significantly decreased the affinity of binding between DHPS and dapsone. In contrast, the T53A and T53I variants may have slightly changes in binding energy and Ki as compared to wild-type DHPS. These changes may reduce the sensitivity of *M. leprae* to dapsone. This finding corresponds to previously reports that showed that the T53I, T53A, and T55V variants mediate resistance to dapsone [8, 10, 11]. A similar report stated that mutations at codons 55 and 53 resulted in dapsone resistance except for T53S [31]. According to the result, then we assumed that the mutation on amino acid number 39 (V39I) might involve in the reducing sensitivity of *M. lepare* to the dapsone.

## 5 Conclusion

A previously unidentified mutation at codon 39 of *folP1* (V39I) was identified from a Papua isolate of *M. leprae*. The binding affinity of this variant for dapsone is predicted to be slightly changed, which may increase the susceptibility of *M. leprae* to dapsone. However, *in vivo* mouse pad studies with are needed to confirm the effect of the V39I mutation on dapsone resistance.

## Declarations

### Author contribution statement

Hana Krismawati, Hotma Hutapea, Antonius Oktavian: Conceived and designed the experiments; Performed the experiments; Wrote the paper.

Widodo, Ratu Fatimah: Performed bioinformatic analysis and interpreted the data; Wrote the paper.

Yustinus Maladan: Conceived and designed the experiments; Performed the experiments; Analyzed and interpreted the data; Wrote the paper.

### Funding statement

This work was supported by the Ministry of Health, Republic of Indonesia.

### Competing interest statement

The authors declare no conflict of interest.

### Additional information

No additional information is available for this paper.
